# Early Miocene reef- and mudflat-associated gastropods from Makran (SE-Iran)

**DOI:** 10.1007/s12542-017-0354-8

**Published:** 2017-06-17

**Authors:** Mathias Harzhauser, Markus Reuter, Tayebeh Mohtat, Werner E. Piller

**Affiliations:** 1 Natural History Museum Vienna, Burgring 7, 1010 Vienna, Austria; 2 Institute of Earth Sciences, NAWI Graz Geocenter, University of Graz, Heinrichstrasse 26, 8010 Graz, Austria; 3 Geological Survey of Iran, Azadi Sq., Meraj-street, Tehran, Iran

**Keywords:** Mollusca, Indian Ocean, Biogeography, Neogene, Makran, Mollusca, Indischer Ozean, Biogeographie, Neogen, Makran

## Abstract

A new gastropod fauna of Burdigalian (early Miocene) age is described from the Iranian part of Makran. The fauna comprises 19 species and represents three distinct assemblages from turbid water coral reef, shallow subtidal soft-bottom and mangrove-fringed mudflat environments in the northern Indian Ocean. Especially the reef-associated assemblage comprises largely new species. This is explained by the rare occurrence of reefs along the northern margin of the Miocene Indian Ocean and the low number of scientific studies dealing with the region. In terms of paleobiogeography, the fauna corresponds well to coeval faunas from the Pakistani Balochistan and Sindh provinces and the Indian Kathiawar, Kutch and Kerala provinces. During the early Miocene, these constituted a discrete biogeographic unit, the Western Indian Province, which documents the near complete biogeographic isolation from the Proto-Mediterranean Sea. Some mudflat taxa might represent examples of vicariance following the Tethys closure. The fauna also displays little connection with coeval faunas from Indonesia, documenting a strong provincialism within the Indo-West Pacific Region during early Miocene times. *Neritopsis gedrosiana* sp. nov., *Calliostoma irerense* sp. nov., *Calliostoma mohtatae* sp. nov. and *Trivellona makranica* sp. nov. are described as new species.

## Kurzfassung

Eine neue Gastropoden-Fauna aus dem Burdigalium (frühes Miozän) des Makrans (SE-Iran) wird beschrieben. Die Fauna umfasst 19 Arten und repräsentiert drei distinkte Vergesellschaftungen: ein Trübwasser-Korallenriff, subtidale Weichböden und Mangroven gesäumte Wattflächen des nördlichen Indischen Ozeans. Die Riff-assoziierte Vergesellschaftung besteht mehrheitlich aus bisher unbeschriebenen Arten. Das erklärt sich aus der geringen Zahl an Korallenriffen im nördlichen Indischen Ozean während des Miozäns und deren unzureichendem Bearbeitungsstand. Paläobiogeographisch zeigt die Fauna deutliche Bezüge zu gleichalten Faunen aus den pakistanischen Provinzen Belutschistan und Sindh sowie zu den westindischen Provinzen Kathiawar, Kutch und Kerala. Während des frühen Miozäns bildeten diese Faunen eine diskrete biogeographische Einheit, die *Western Indian Province*, die eine nahezu vollständige biogeographische Isolation vom Proto-Mittelmeer zeigt. Die morphologische Ähnlichkeit von einigen Arten aus der Wattflächenvergesellschaftung mit Arten aus der WIP/MIP ist vermutlich ein Beispiel für Vikarianz infolge der Tethys-Schließung. Zu gleichalten indonesischen Faunen sind die biogeographischen Bezüge ebenfalls sehr gering, was auf einen starken Provinzialismus innerhalb der *Indo*-*West Pacific Region* während des frühen Miozäns hinweist. *Neritopsis gedrosiana* sp. nov., *Calliostoma irerense* sp. nov., *Calliostoma mohtatae* sp. nov. and *Trivellona makranica* sp. nov. werden als neue Arten beschrieben.

## Introduction

The knowledge on the evolution and composition of the Miocene mollusc fauna of the Indian Ocean is still spotty due to the very limited fossil record. During the last years, Miocene gastropod assemblages from the Sultanate of Oman, coastal Tanzania, Kutch (NW-India) and Kerala (SW-India) have been described and revised by Harzhauser (2007, 2009, 2014) and Harzhauser et al. (2009) and Jain (2014). These revisions document a biogeographically subdivided pattern in the Indian Ocean during the early Miocene with no or negligible faunal exchange with the Proto-Mediterranean region and moderate faunistic relations with the coeval Indonesian faunas. An area of comparatively homogenous faunistic composition stretched from Balochistan and Sindh in Pakistan via Gujarat in NW-India down to Kerala in SW-India for which Harzhauser (2007) introduced the biogeographic term Western Indian Province (IWP).

First descriptions of the faunas of the Balochistan and Sindh provinces and the Indian Kathiawar and Kutch provinces in Gujarat date back to the 19th century and start with the contributions by Sowerby (1840) and Archiac and Haime (1853). Both monographs deal with an assortment of species from differing stratigraphic levels and especially Archiac and Haime (1853) partly mixed Miocene specimens with Cretaceous or Paleocene ones, without providing locality data. Moreover, many taxa were based on unidentifiable internal casts. Fedden (1880) tried to assign the taxa of Archiac and Haime (1853) to discrete stratigraphic levels, but several species remained dubious. The first modern and comprehensive description, treating Oligocene and early Miocene mollusc faunas from Balochistan, Sindh, Kathiawar and Kutch, was provided by Vredenburg (1925, 1928). Like Fedden (1880), he recognized that the occurrences from the so-called Gaj beds are of early Miocene age. Since then, only few additional contributions were published by Harzhauser et al. (2009), Kulkarni et al. (2010) and Jain (2014), focusing on localities in the Indian Gujarat; Iqbal (1980) discussed Pakistani occurrences.

Whilst the mollusc faunas from the Pakistani part of the Makran were included by Vredenburg (1925, 1928) the western extension in Iran remained so far unstudied. These, however, represent the north-westernmost fossiliferous geological records of this part of the Indian Ocean, for which McCall et al. (1994) coined the term Proto-Persian Gulf. The area is close to the suspected Gomphotherium-landbridge, that separated the Proto-Mediterranean Sea from the Indian Ocean during Burdigalian times (Rögl 1998; Harzhauser et al. 2007; Reuter et al. 2007). The early Miocene mollusc assemblages from the Iranian Makran might thus be expected to reflect the coincident biogeographic separation of marine faunas. Whereas the early Miocene coral fauna of the Iranian Makran is fairly well studied (McCall et al. 1994; Ghaedi et al. 2016), the mollusc fauna of the Proto-Persian Gulf have not attracted any attention so far. Therefore, herein we describe gastropod assemblages collected along a long section of the Band-e-Chaker Formation, which comprises siliciclastic peri-tidal deposits and coral-reef carbonates, which formed on the Makran shelf during the Burdigalian (Ghaedi et al. 2016). These assemblages allow a realistic estimate of the effect of the Tethyan closure on faunal exchange between the Proto-Mediterranean Sea and the northern Indian Ocean.

## Geological setting

**Fig. 1:**
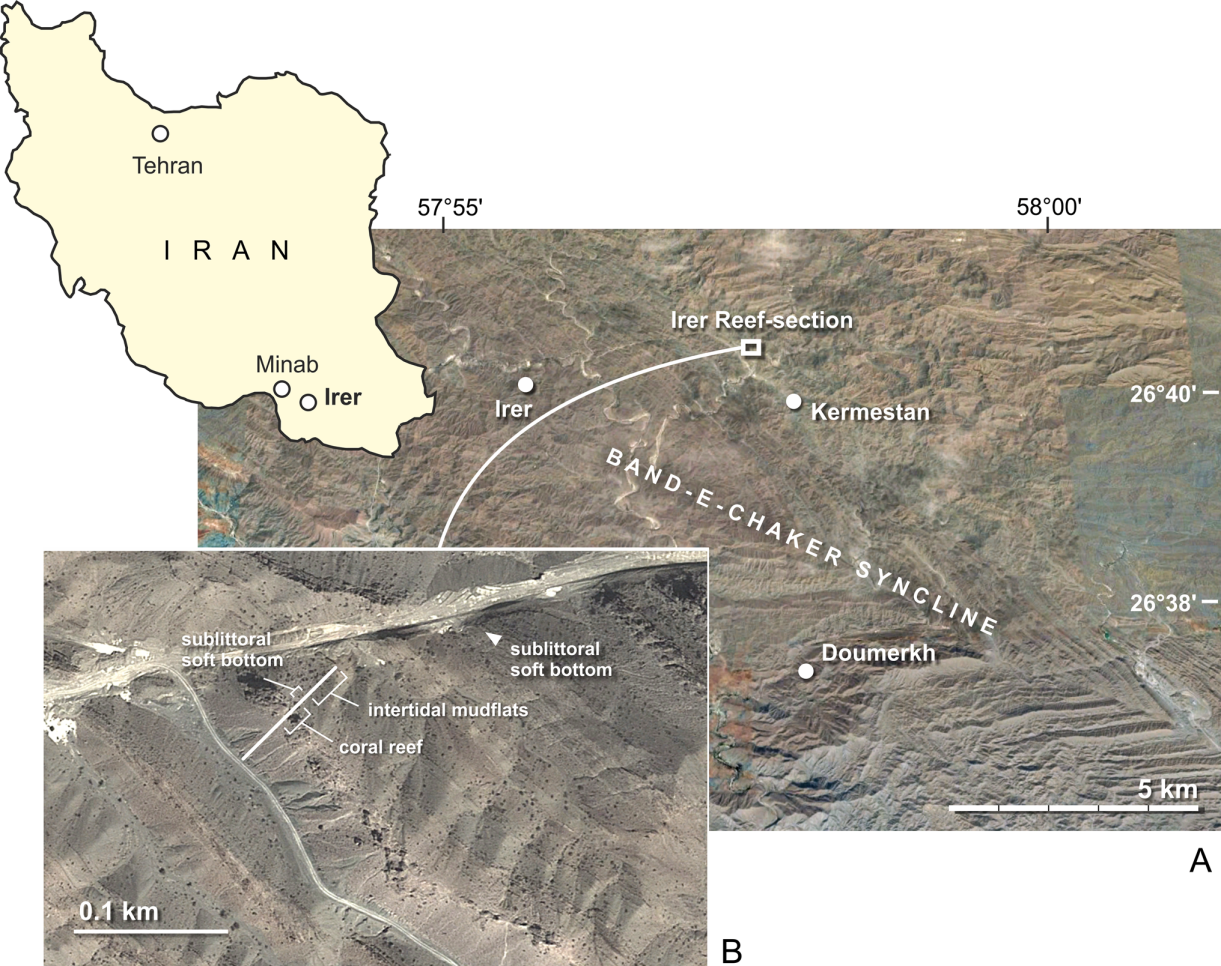
Geographic position of the investigated section at the eastern flank of the Band-e-Chaker Syncline (**a**) and detailed view of the succession (**b**) indicating the occurrences of the three gastropod assemblages and their assumed paleoenvironment (maps generated with Google Earth, Image © 2016 Digital Globe; image taken in 2012)

The Makran mountain range in southeastern Iran and southwestern Pakistan is the onshore part of a ~400 km wide and up to 7.5 km thick oceanic accretionary wedge. It has developed throughout the Cenozoic due to frontal accretion and underplating of trench fill sediments at the Makran Subduction Zone under the condition of high sediment input from the Indus River and extreme erosion of the inner parts of the growing accretionary prism (Platt et al. 1985; Kopp et al. 2000; Schlüter et al. 2002; Ellouz-Zimmermann et al. 2007a, b; Smith et al. 2012). From the Palaeocene to middle Miocene, a prograding clastic wedge evolved, including successions shallowing upward from deep-water turbidites into slope and shelf deposits (Crimes and McCall 1995; Burg et al. 2008). The herein presented gastropod fauna was mainly collected along a continuous stratigraphic section in marginal and shallow marine mixed siliclastic-carbonate sediments of early Miocene age that are exposed near Irer village at the eastern flank of the Band-e-Chaker Syncline in the Western Makran (N 26°40′55.26′′, E 057°56′1.72′′; Irer locality of Ghaedi et al. 2016) (Fig. 1). Additional gastropods derived from sandstone beds and dark grey phytoclastic marls, which are exposed below (sandstones) and above (phytoclastic marls) the measured section. The Irer section starts with a 44-m-thick alternation of marls and thin channel-fill deposits including sandstones and sandy grain-/packstones with *Ditrupa* coquinas. The latter contain some shells of potamidids and small turritellids. Wave ripples are commonly preserved at the top of thin sandstone beds. Intercalated within this alternation are several deposits of dark grey marl with abundant plant debris, in situ rootlets, *Terebralia* and certain septarian levels. A bed of pedogenic carbonate and a stromatolith were also found in contact to such a phytoclastic marl at the base of the investigated lithological succession. This facies association points to extensive tidal flats, which have been colonized by mangroves in the upper part. The next sedimentary unit is 11 m thick and consists of marls, calcareous marls and argillaceous limestones, which formed in shallow subtidal environments. It is conformably overlain by marly coral limestones (13 m thick) with characteristic sheet and platestone growth fabrics (sensu Insalaco 1998) which have been interpreted as turbid water reef (Ghaedi et al. 2016). The Irer section continues with an at least 20-m-thick succession of grey-green marls with thin, randomly distributed interbeds of calcareous marl that is devoid of macrofossils. In terms of lithostratigraphy, the upper part of the Irer section, including the coral limestones and the overlying grey-green marls, was assigned to the Burdigalian Band-e-Chaker Formation by Ghaedi et al. (2016). This Burdigalian age is biostratigraphically confirmed by the presence of the foraminifer *Miogypsina globulina* in the coral-limestone unit (Ghaedi et al. 2016). The underlying sedimentary succession was tentatively assigned to the Aquitanian Dehirdan Formation by Ghaedi et al. (2016) without providing any arguments for this separation. The gradual sedimentary contact as well as the almost identical vertical successions of facies below and above the coral limestone unit does, however, not support this lithostratigraphic separation. Mollusc faunas in mangrove-associated mudflat facies are also not different below and above the coral limestone unit. Furthermore, the Irer section shows a gradual deepening by the transition from intertidal mudflats via shallow marine soft-bottom environments to a coral reef but the Aquitanian/Burdigalian boundary is marked by a third order sea-level drop (Aq3/Bur1 of Hardenbol et al. 1998). For these reasons. we do not follow Ghaedi et al. (2016) and place the Irer section entirely in the Band-e-Chaker Formation of Burdigalian age.

## Material

The material was collected during fieldwork in February 2017 in cooperation with the Geological Survey of the Islamic Republic Iran. All specimens are stored in the paleontological collection of the Natural History Museum Vienna (NHMW).

### Systematic paleontology

Class **Gastropoda** Cuvier, 1795

Subclass **Neritimorpha** Golikov and Starobogatov, 1975

Order **Cycloneritimorpha** Bandel and Frýda, 1999

Superfamily **Neritopsoidea** Gray, 1847

Family **Neritopsidae** Gray, 1847

Genus ***Neritopsis*** Grateloup 1832

*Type species. Neritopsis moniliformis* Grateloup 1832; by monotypy. Lower Miocene Aquitaine Basin, France.

***Neritopsis gedrosiana*** Harzhauser sp. nov.

Figure 2a, b

**Fig. 2:**
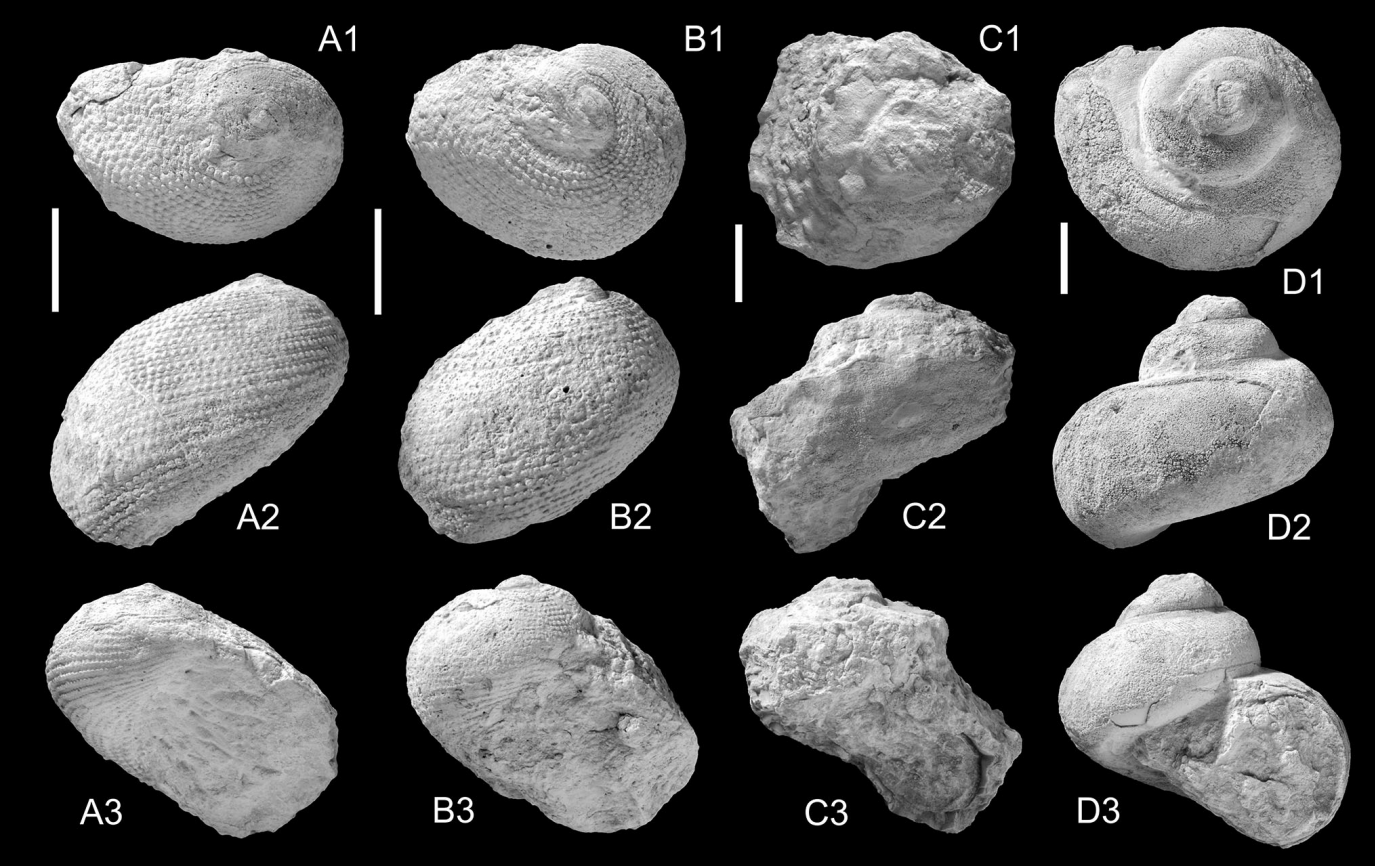
Neritopsidae, Angariidae, Turbinidae. **a**, **b** *Neritopsis gedrosiana* Harzhauser sp. nov., **a** Holotype, NMHW 2017/0033/0001, **b** paratype NMHW 2017/0033/0002; **c** *Angaria* cf. *delphinus* (Linnaeus, 1758), NMHW 2017/0033/0005; **d** *Turbo* cf. *petholatus* Linnaeus, 1758, NMHW 2017/0033/0014. *Scale bar*  10 mm

*Etymology.* Referring to the ancient name Gedrosia for the Makran area.

*Holotype.* NMHW 2017/0033/0001, height: 26.1 mm, diameter: 29.2 mm (Fig. 2a).

*Paratype.* NMHW 2017/0033/0002, height: 22.5 mm, diameter: 26.7 mm (Fig. 2b).

*Paratype.* NMHW 2017/0033/0003, height: 35.5 mm, diameter: 42.5 mm.

*Paratype.* NMHW 2017/0033/0004 (compressed specimen): diameter: 36 mm.

*Locality and horizon.* Close to Irer village (N 26°40′55.26′′, E 057°56′1.72′′), Makran, Islamic Republic of Iran; reefal limestone of the Band-e-Chaker Formation, Burdigalian, early Miocene.

*Diagnosis.* Large *Neritopsis* with densely spaced spiral rows of densely spaced, delicate beads separated by faint secondary spiral rows of beads along periphery.

*Description.* Large for genus, wider than high, with weakly protruding spire consisting of c. two convex whorls; protoconch unknown. Last whorl large, rapidly widening, regularly convex at periphery, with narrow sutural ramp and distinct suture. Sculpture consisting of c. 30–35 densely spaced spiral rows of densely and regularly spaced rounded beads, which do not form an axial pattern. Spiral rows separated by narrow spiral grooves on adapical part of whorl but widening along periphery. Weak secondary spiral cords with faint beads may be intercalated between primary cords along periphery. Aperture subcircular, obscured by sediment; outer lip strongly prosocline, thin. Umbilicus concealed, indicated by shallow depression.

*Remarks.* No *Neritopsis* species has been described so far from the Miocene of the Indian Ocean. The coeval Western Tethyan/Proto-Mediterranean *Neritopsis moniliformis* Grateloup 1832 is much smaller, more spherical and has coarser sculpture (see Lozouet et al. 2001). The extant *Neritopsis radula* (Linnaeus, 1758), which originated in the IWP Region during the early Miocene (Ladd 1966), differs in its higher spire, coarser sculpture and less flaring last whorl. The extant *Neritopsis richeri* Lozouet, 2009, from French Polynesia, differs in its more prominent secondary spiral cords, the coarser beads and the smaller size. Extant *Neritopsis* species are typically found in submarine caves (Kano et al. 2002; Lozouet 2009). Comparable habitats may thus be expected for this Miocene species from Iran.

*Stratigraphic and geographic distribution.* Only known from the type locality.

Subclass **Vetigastropoda** Salvini-Plawen, 1980

Superfamily **Angarioidae** Gray, 1857

Family **Angariidae** Gray, 1857

Genus ***Angaria*** Röding, 1798

*Type species. Turbo delphinus* Linnaeus, 1758; subsequent designation by Fischer (1878). Recent, Indo-West Pacific.

***Angaria*** cf. ***delphinus*** (Linnaeus, 1758)

Figure 2c

cf. *1758 *Turbo delphinus* Linnaeus: p. 764.

cf. 1966 *Angaria delphinus* (Linnaeus)—Ladd: p. 42, pl. 5, Figs. 29–34.

cf. 1996 *Angaria* (*Angaria*) *delphinus* (Linnaeus, 1758)—Robba: p. 290, Fig. 8 (cum syn.).

cf. 2008 *Angaria delphinus* (Linnaeus, 1758)—Monsecour: p. 228, pl. 59, Figs. 3, 4.

*Material.* One specimen (NMHW 2017/0033/0005).

*Measurements.* height: 33.0 mm, diameter: 35.1 mm.

*Description.* Solid shell with broad, gradate spire comprising about five teleoconch whorls. Early spire whorls strongly abraded; last spire whorl angulated, with nearly flat sutural ramp. Sculpture consisting of broad, knob-like swellings close below upper suture and prominent triangular nodes along shoulder. Last whorl sloping in lower direction resulting in oblique suture. Low angled sutural ramp with four spiral rows of irregular blunt nodes; shoulder with wide spaced spiny nodes, pointing slightly in apical direction. Periphery flat, weakly contracting; transition into convex base angulated. Base covered by several spiral cords with broad nodes, which become scaly in the circum-umbilical area; umbilicus covered by sediment.

*Remarks.* The sculpture of the specimen is partly abraded, which causes uncertainties about the strengths and lengths of the various nodes. The overall morphology agrees well with *Angaria delphinus*, which is widespread in the IWP-Neogene.

*Stratigraphic and geographic distribution.* Fossils identified as *Angaria delphinus* are recorded from the lower Miocene of Gujarat and Kerala in India (Dey 1961; Kulkarni et al. 2010), from the lower to upper Miocene and Pliocene of Indonesia and Borneo (Van Regteren Altena 1938; Beets 1950; Robba 1996), the Miocene of Guam and Fiji (Ladd 1966), the Pliocene of Japan (Tomida 2005) and the Pleistocene of Timor and Sumba (Tesch 1920; Robba 1996). The extant species is found today in the Southwest Pacific and Australia.

Superfamily **Trochoidea** Rafinesque, 1815

Family **Turbinidae** Rafinesque, 1815

Genus ***Turbo*** Linnaeus, 1758

*Type species. Turbo petholatus* Linnaeus, 1758; subsequent designation by Montfort (1810). Recent, Indo-West Pacific.

***Turbo*** cf. ***petholatus*** Linnaeus, 1758

Figure 2d

cf. *1758 *Turbo petholatus* Linnaeus: p. 762.

cf. 1966 *Turbo* (*Turbo*) *petholatus* Linnaeus – Ladd: p. 47, pl. 7, Figs. 21–22.

cf. 2008 *Turbo petholatus* Linnaeus, 1758—Kreipl and Alf: p. 260, pl. 75, Figs. 4–8.

*Material.* Three specimens (NMHW 2017/0033/0014).

*Measurements.* height: 29.5 mm, diameter: 44.1 mm; height: 37 mm (compressed): diameter: 50.5 mm.

*Description.* Medium-sized turbinid with moderately high spire; spire whorls convex with faint shoulder; last whorl and base regularly convex, rapidly increasing in diameter. Shell surface smooth aside from prosocline growth lines and regularly spaced prosocline grooves, most prominent below suture and fading out below periphery. Weak spiral cord along upper suture. Umbilicus covered, indicated by shallow depression. Aperture circular with thickened and slightly reflected basal peristome.

*Remarks.* The specimens are highly reminiscent of the extant *Turbo petholatus*. The small size and the deeper umbilical depression might indicate that the Iranian Burdigalian shells represent a separate species but the poor preservation does not allow a clear identification.

*Stratigraphic and geographic distribution.* The earliest record of *Turbo petholatus* is documented from the early Miocene of Fiji (Ladd 1966). The extant species became widespread during the Pliocene and Pleistocene in the entire IWP-region (Tesch 1920; MacNeil 1961; Ladd 1982; Mimoto and Nakao 2010).

Family **Calliostomatidae** Thiele, 1924 (1847)

Genus ***Calliostoma*** Swainson, 1840

*Type species. Trochus conulus* Linnaeus, 1758; subsequent designation by Herrmannsen (1846). Recent, Mediterranean Sea.

***Calliostoma irerense*** Harzhauser sp. nov.

Figure 3a–c

**Fig. 3:**
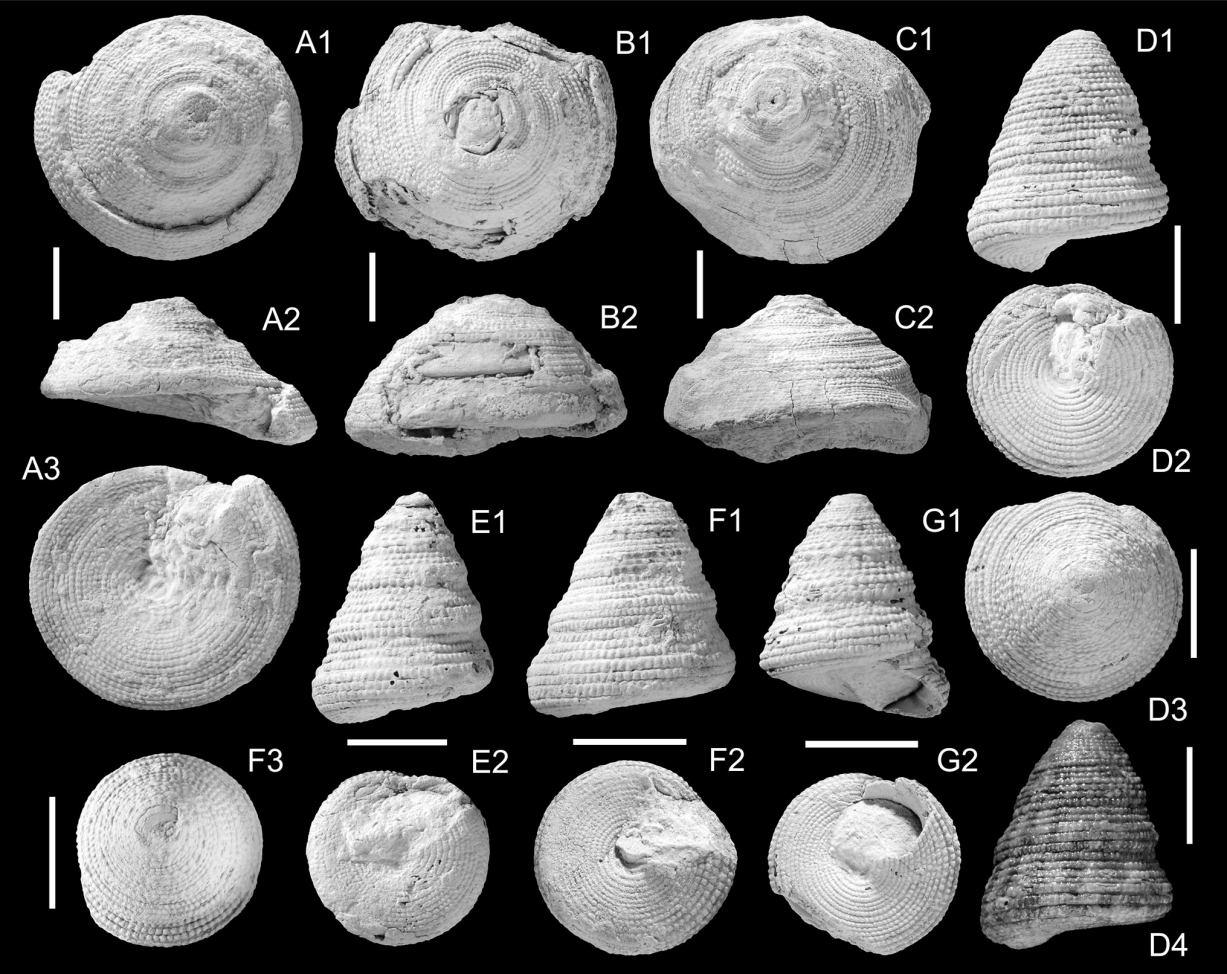
Calliostomatidae. **a**–**c** *Calliostoma irerense* Harzhauser sp. nov., **a** Holotype, NMHW 2017/0033/0006, **b** paratype, NMHW 2017/0033/0007, **c** paratype, NMHW 2017/0033/0008; **d**–**g** *Calliostoma mohtatae* Harzhauser sp. nov., **d** holotype, NMHW 2017/0033/0009, *D4* showing the color pattern, **e** Paratype, NMHW 2017/0033/0010, **f** paratype, NMHW 2017/0033/0011, **g** paratype, NMHW 2017/0033/0012. * Scale bar*  10 mm

*Etymology.* Referring to the village Irer close to the section.

*Holotype.* NMHW 2017/0033/0006, height: 23.5 mm, diameter: 39.4 mm (Fig. 3a).

*Paratype.* NMHW 2017/0033/0007, height: 21.7 mm, diameter: 42.4 mm (Fig. 3b).

*Paratype.* NMHW 2017/0033/0008, height: 26.7 mm, diameter: 42.5 mm (Fig. 3c).

*Locality and horizon.* Close to Irer village (N 26°40′55.26′′, E 057°56′1.72′′), Makran, Islamic Republic of Iran; reefal limestone of the Band-e-Chaker Formation, Burdigalian, early Miocene.

*Diagnosis.* Large depressed trochiform shell flange-like periphery and sculpture of 10–11 densely spaced, beaded spiral cords; flat base and narrow, open umbilicus.

*Description.* Large, broad, depressed trochiform, solid shell; early spire slightly coeloconoid, later low cyrtoconoid. Protoconch and early teleoconch whorls not preserved. Early spire whorls with shallow concavity in lower third causing a flange-like outline of periphery. Concavity disappears gradually during growth. Sculpture consisting of 10–11 densely spaced and delicately beaded spiral cords; beads differ slightly in strength on individual spiral cords and do not form any axial pattern. Interspaces between spiral cords smooth, very narrow. Suture weakly incised. Periphery of fully grown specimens weakly rounded passing into flat base, covered by c. 20 beaded spiral cords; umbilicus open, narrow.

*Remarks.* This species is characterized by its broad trochiform outline and large size. No comparable species was described so far from the Miocene of the Indian Ocean. “*Trochus” kathiawarensis* Jain, 2014, from the Gaj beds of Gujarat (India), is superficially similar, but differs in its much higher spire and the coarse sculpture. Among extant species, *Calliostoma selecta* (Dillwyn, 1817), from New Zealand, and the western Atlantic *Calliostoma benedicti* (Dall, 1889) are somewhat reminiscent of the Iranian species but differ in the closed umbilicus (*C. selcta*) and the smaller size and coeloconoid spire (*C. benedicti*). Its autochthonous occurrence within the reefal limestones, suggests that this species was associated with corals.

*Stratigraphic and geographic distribution.* Only known from the type locality.

***Calliostoma mohtatae*** Harzhauser sp. nov.

Figure 3d–g

*Etymology.* In honor of Tayebeh Mohtat (Geological Survey, Tehran, Islamic Republic of Iran).

*Holotype.* NMHW 2017/0033/0009, height: 25.4 mm, diameter: 20.6 mm (Fig. 3d).

*Paratype.* NMHW 2017/0033/0010, height: 23.9 mm, diameter: 18.6 mm (Fig. 3e).

*Paratype.* NMHW 2017/0033/0011, height: 20.7 mm, diameter: 17.9 mm (Fig. 3f).

*Paratype.* NMHW 2017/0033/0012: height: 21.2 mm, diameter: 17.6 mm (Fig. 3g).

*Additional material.* One hundred and twenty specimens (NMHW 2017/0033/0013).

*Locality and horizon.* Close to Irer village (N 26°40′55.26′′, E 057°56′1.72′′), Makran, Islamic Republic of Iran; reefal limestone of the Band-e-Chaker Formation, Burdigalian, early Miocene.

*Diagnosis.* Medium-sized conical trochiform shell; early spire whorls with weak subsutural concavity and carina at lower suture. Sculpture consisting of six densely spaced and densely beaded spiral cords; flat base with beaded spiral cords, nearly closed umbilicus.

*Description.* Medium-sized, conical trochiform shell of about 8–9 teleoconch whorls. Protoconch unknown. Early spire conical to slightly cyrtoconoid passing into conical teleoconch with an angle typically ranging around 40° (but attaining up to 50°). Early spire whorls always strongly abraded, bearing four delicately beaded spiral cords and a fifth prominent adsutural cord with protruding nodes, forming a carina at lower suture; carina restricted to one or two whorls; later teleoconch whorls covered by six densely spaced and densely beaded spiral cords; beads are often subquadratic on last two teleoconch whorls and especially in lower half of whorl. Upper half of whorl often coinciding with a shallow concavity; lower half often slightly bulgy with more prominent spiral cords. Two narrow, weakly beaded secondary spiral cords may be intercalated between lower three primary cords on last whorl. Periphery narrowly rounded passing into flat base with 11–13 beaded spiral cords. Beads on base are prominent close to periphery and become gradually weaker towards umbilicus. Umbilicus reduced to narrow chink, smooth, demarcated from base by rim-like spiral cord. Inner lip narrow, slightly thickened. Outer lip strongly prosocline, rapidly thinning, slightly flaring, with a single weak spiral ridge close to the base inside.

Some specimens display remnants of color patterns consisting of small, vaguely axially arranged speckles on the base and broad, opisthocline axial bands on the spire whorls separated by slightly wider light bands.

*Remarks.* The species is somewhat variable in shape ranging from slender to more bulky shells and in more or less bulgy outline of the lower half of the whorls. *Calliostoma jujubiniforme* (Martin, 1884), from the Eocene? of Indonesia, is highly reminiscent of the Iranian species concerning size, outline and the carinate early teleoconch whorls but lacks the beaded sculpture (see Leloux and Wesselingh 2009). *Calliostoma dyscritum* Cossmann, 1910, from the Pliocene of S-India, and *C. cosijni* Van Regteren Altena, 1938, from the Pliocene of Indonesia, might be related but are smaller and lack the bulgy outline of the spire whorls. Several extant IWP-species, such as *Calliostoma suduirauti* Bozzetti, 1997, *C. scobinatum* (Reeve, 1863), *C. swinneni* Poppe, Tagaro and Dekker, 2006 and *C. aliguayensis* Poppe, Tagaro and Dekker, 2006, develop a comparable sculpture and outline but differ in their smaller size and the deep water habitat (Poppe and Tagaro 2008).

This is the most abundant mollusc species in the Irer-reef and is frequently found in situ between the corals. Therefore, it doubtlessly represents a reef inhabitant.

*Stratigraphic and geographic distribution.* Only known from the type locality.

Subclass **Caenogastropoda** Cox, 1960

Unassigned order (formerly as Architaenioglossa Haller, 1892, which is invalid according to Harasewych et al. 1998)

Superfamily **Ampullinoidea** Cossmann, 1918

Family **Ampullinidae** Cossmann, 1918

Genus ***Cernina*** Gray, 1840

*Type species. Natica fluctuata* Sowerby 1825; subsequent designation by Gray (1847). Recent, Philippines.

***Cernina carlei*** (Finlay, 1927)

1840 *Natica callosa* J.C. Sowerby, explanation of plates (no page number), pl. 26 Fig. 3 [non *Natica callosa* Scopoli 1777, non Cristofori and Jan 1832].

*1927 *Natica carlei* Finlay: p. 498.

2009 *Globularia carlei* (Finlay)—Harzhauser et al.: p. 337, Fig. 2c, d (cum syn.).

2014 *Cernina carlei* (Finlay, 1927)—Harzhauser: p. 89, pl. 3, Figs. 6–8 (cum syn.).

Non 2010 *Globularia* (*Globularia*) *carlei* (Finlay 1927) Dey, 1961—Kulkarni et al.: p. 327, Fig. 3b.

*Material.* One specimen (NMHW 2017/0033/0015) and numerous specimens from the marly sandstone underlying the reef carbonates at Irer (field observation).

*Measurements.* height: 24.5 mm, diameter: 25.8 mm.

*Remarks.* This species was discussed in detail by Harzhauser et al. (2009, 2014). Beu and Marshall (2011) consider *Cernina carlei* to be a synonym of the extant *Cernina fluctuata* (Sowerby, 1825). In our opinion, the low spire and small size of the specimens from the lower Miocene Gaj beds of Iran, Pakistan and India justify a separation on species level as already discussed by Vredenburg (1928). The specimen from Gujarat, identified by Kulkarni et al. (2010) as *Globularia carlei*, differs in the gradate spire and might rather represent a naticid.

The species is quite abundant in the lagoonal sandstones underlying the reefal carbonates but is absent from the reef and mudflat assemblages.

*Stratigraphic and geographic distribution. Cernina carlei* is known from the Burdigalian of the Kutch Basin in NW-India (Harzhauser et al. 2009), Kerala in SW-India (Harzhauser 2014) and the Iranian Makran (this paper).

Unassigned order

Superfamily **Cerithioidea** Fleming, 1822

Family **Potamididae** Adams and Adams, 1853

Genus ***Terebralia*** Swainson, 1840

*Type species. Strombus palustris* Linnaeus, 1758; subsequent designation by Sacco (1895). Recent, Indo-West Pacific.

***Terebralia gajensis*** Vredenburg, 1928

Figure 4a–c

**Fig. 4:**
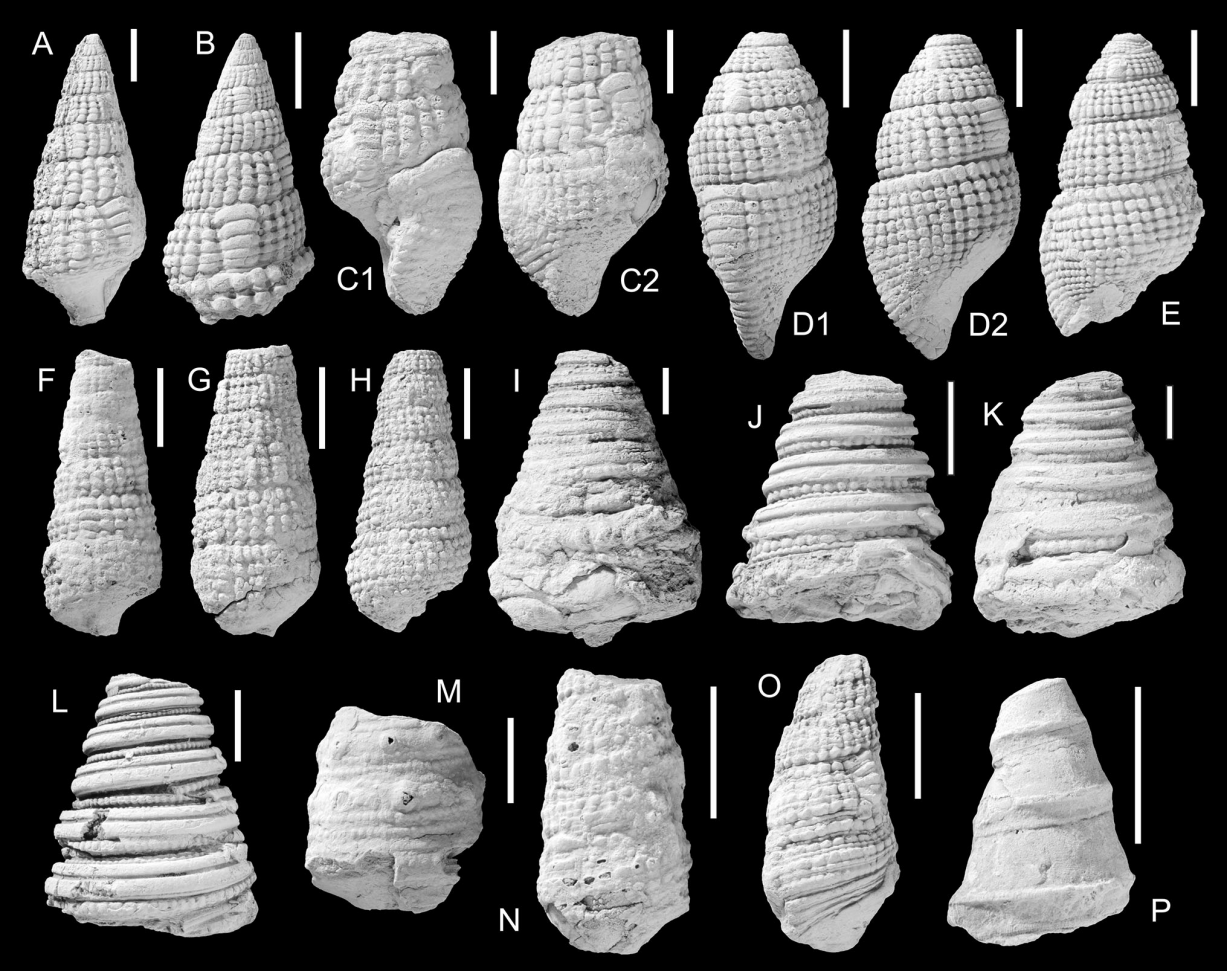
Potamididae, Cerithiidae, Turritellidae. **a**–**c** *Terebralia gajensis* Vredenburg, 1928, **a** NMHW 2017/0033/0016, **b** NMHW 2017/0033/0017, **c** NMHW 2017/0033/0018; **d**, **e** *Terebralia miosulcata* Vredenburg, 1928, **d** NMHW 2017/0033/0019, **e** NMHW 2017/0033/0020; **f**–**h** *Terebralia sublignitarum* Vredenburg, 1928, **f** NMHW 2017/0033/0023, **g** NMHW 2017/0033/0024, **h** NMHW 2017/0033/0025; **i**–**l** *Campanilopsis pakistanica* (Eames, 1950), **i** NMHW 2017/0033/0026, **j** NMHW 2017/0033/0027, **k** NMHW 2017/0033/0028a, **l** NMHW 2017/0033/0028b; **m** *Vicarya verneuili* (Archiac, 1851), NMHW 2017/0033/0029); **n** *Cerithium* sp., NMHW 2017/0033/0030, **o** *Cerithium* sp., NMHW 2017/0033/0031; **p** *Zaria angulata* (Sowerby, 1840), NMHW 2017/0033/0033). *Scale bar*  10 mm

*1928 [*Terebralia bidentata* Dèfr. var.] *gajensis* Vredenburg: p. 367, pl. 16, Figs. 9–10, pl. 18, Fig. 1.

1980 *Terebralia bidentata* Defr. ssp. *gajensis* Vredenburg—Iqbal: p. 40, pl. 33, Fig. 1.

*Material.* Five specimens, NMHW 2017/0033/0016 (Fig. 4a), NMHW 2017/0033/0017 (Fig. 4b), NMHW 2017/0033/0018 (Fig. 4c).

*Measurements.* height: 54.3 mm, diameter: 23.9 mm (Fig. 4a); largest specimen: diameter: 27.9 mm.

*Description.* Large *Terebralia* with conical spire; apical angle ranging around 30°–33°. Protoconch and early teleoconch whorls unknown; following spire whorls straight-sided with densely spaced axial ribs separated by deep spiral grooves, resulting in four spiral rows of subquadratic beads. Spiral grooves and axial interspaces between ribs become gradually wider resulting in rather wide-spaced subquadratic to slightly spirally elongated beads on last two teleoconch whorls. Axial ribs weakly opisthocline on weakly convex penultimate and last whorl. Incised suture wavy at intersection with blunt and broad varices. Last whorl moderately elongated with very prominent varix. Aperture not preserved in the available material.

*Remarks.* This species is an Indo-West Pacific pendant of the Western Tethyan/Proto-Mediterranean *Terebralia lignitarum* (Eichwald, 1830), from which it differs in its more obtuse spire, the comparatively blunter sculpture and the broader somewhat expanded last whorl (see Landau et al. 2013 for a discussion on the nomenclature of *Terebralia lignitarum*/*bidentata/duboisi*).

*Stratigraphic and geographic distribution.* Burdigalian of Irer in Iran (Makran) and lower Miocene Gaj beds of Eri Hill in Sindh (Pakistan) (Vredenburg 1928).

***Terebralia miosulcata*** Vredenburg, 1928

Figure 4d, e

*1928 *Terebralia miosulcata* n. sp. Vredenburg: p. 366, pl. 16, Figs. 1–8, pl. 17, Figs. 10–11, 13.

*Material.* Eleven specimens, NMHW 2017/0033/0019 (Fig. 4d), NMHW 2017/0033/0020 (Fig. 4e), NMHW 2017/0033/0021 (1 spec.), NMHW 2017/0033/0022 (eight spec).

*Measurements.* largest specimen: height: 46.0 mm, diameter: 20.5 mm.

*Description:* Medium-sized, stout, pupoid shells. Protoconch and early teleoconch whorls unknown. Early spire narrowly conical passing rapidly in broad cyrtoconoid spire; teleoconch whorls convex, suture moderately impressed. Sculpture consisting of densely spaced axial ribs (up to 35 on last whorl) incised by deep spiral grooves resulting in prominent subquadratic beads. Axial ribs orthocline to weakly opisthocyrt. Four spiral rows of beads on upper spire whorls; six spiral rows on penultimate whorl and about 18 spiral rows on last whorl and base. Uppermost spiral cord usually separated by a broader spiral groove. Last whorl moderately convex, passing rapidly into base; aperture strongly elongate. One prominent varix on each whorl being most prominent on last whorl close to aperture.

*Remarks.* Differs from the extant *Terebralia sulcata* in the conspicuous cyrtoconoid early teleoconch and the higher last whorl. The pupoid outline allows a clear separation from the co-occurring *Terebralia gajensis* and *T. sublignitarum*.

*Stratigraphic and geographic distribution.* Known from the Burdigalian of Irer in Iran (Makran) and WSW of Ban in Pakistan (Makran). The Pakistani occurrence was reported by Vredenburg (1928) from the Talar beds, which overlie the lower Miocene Gaj beds and underlie the Pliocene Gwadar beds. Therefore, a middle to late Miocene age can be assumed for the Pakistani record.

***Terebralia sublignitarum*** Vredenburg, 1928

Figure 4f–h

*1928 *Terebralia sublignitarum* n. sp. Vredenburg: p. 368, pl. 18, Figs. 2–5.

**Fig. 5:**
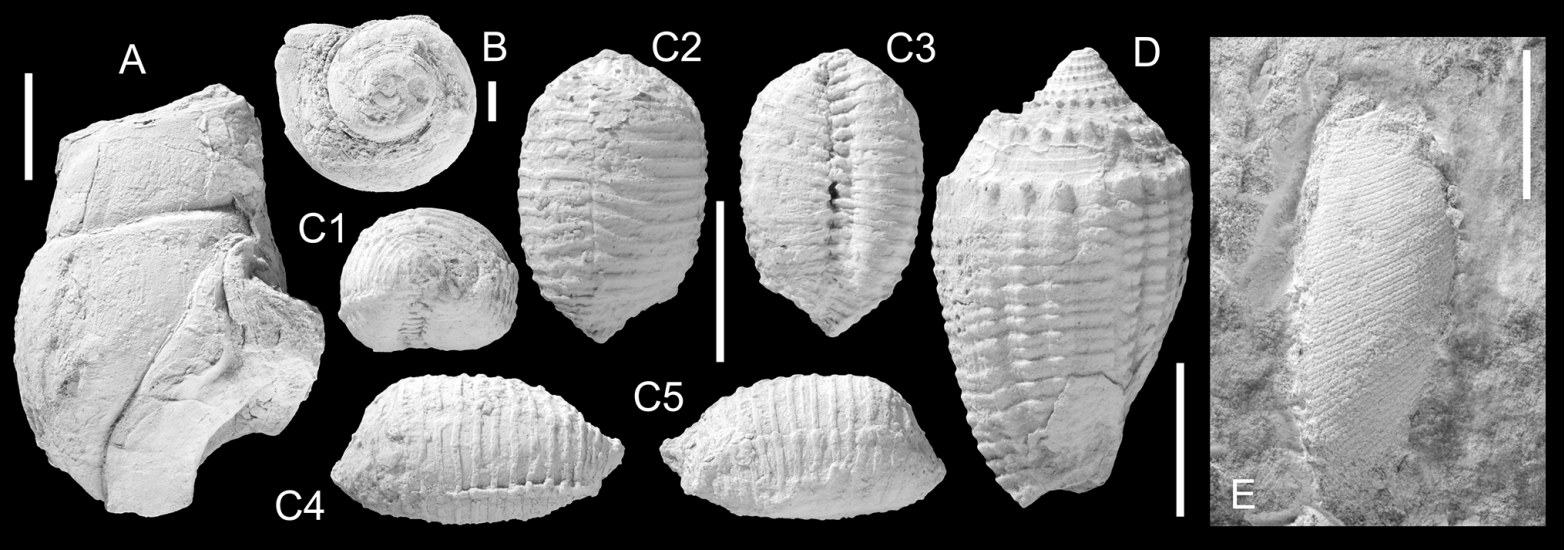
Rostellariidae, Triviidae, Melongenidae, Volutidae, Scaphandridae. **a** *Tibia* cf. *indica* Dey, 1961, NMHW 2017/0033/0034; **b** *Melongena* cf. *lainei* (Basterot 1825), NMHW 2017/0033/0037; **c** *Trivellona makranica* Harzhauser sp. nov., Holotype, NMHW 2017/0033/0035; **d** *Athleta* (*Volutospina*) *sykesi* (Archiac and Haime 1853), NMHW 2017/0033/0038; **e** *Scaphander javanus* (Martin, 1879), NMHW 2017/0033/0039. *Scale bar* 10 mm

*Material.* Ten specimens, NMHW 2017/0033/0023 (Fig. 4f), NMHW 2017/0033/0024 (Fig. 4g), NMHW 2017/0033/0025 (Fig. 4h + seven spec.).

*Measurements.* largest (incomplete) specimen: height: >40 mm, diameter: 17.2 mm.

*Description.* Medium-sized, slender conical shells with an apical angle of 20°–25°. Protoconch and early teleoconch whorls unknown; spire whorls straight sided with weakly incised suture. Sculpture consisting of densely spaced axial ribs incised by narrow spiral grooves resulting in four rows of rounded beads on spire whorls passing into nearly quadratic beads on last teleoconch whorls. Narrow secondary spiral cords may appear on penultimate and last whorl bearing weak, spirally elongate beads. Varices inconspicuous, only slightly more prominent on last whorl. Aperture not preserved in the available material.

*Remarks.* The slender conical outline, low height of spire whorls, weakly incised suture and weak varices allow a clear separation from the co-occurring *Terebralia gajensis* and *T. miosulcata*. As pointed out by Vredenburg (1928) this species is reminiscent of the Western Tethyan/Proto-Mediterranean *Terebralia duboisi* (Hörnes, 1855), which differs in its larger size, the somewhat gradate spire, the absence of secondary spiral cords and the continuous spiral cord connecting the beads (see Landau et al. 2013 for a discussion on the nomenclature of *Terebralia lignitarum*/*bidentata/duboisi*).

*Stratigraphic and geographic distribution.* Burdigalian of Irer in Iran (Makran) and middle to upper Miocene Talar beds WSW of Ban in Pakistan (Makran) (Vredenburg 1928).

Genus ***Campanilopsis*** Chavan, 1949

*Type species. Cerithium ceres* Orbigny 1852; by original designation. Oligocene, France.

***Campanilopsis pakistanica*** (Eames, 1950) comb. nov.

Figure 4i–l

1853 *Cerithium subtrochleare* sp. nov. Archiac and Haime: p. 302, pl. 29, Fig. 2 (non-*Cerithium subtrochleare* Orbigny, 1852).

*1950 *Telescopium pakistanicum* Eames: p. 87 (nov. nom. pro *Cerithium subtrochleare* Archiac and Haime 1853, non Orbigny, 1852).

*Material.* Eleven fragmentary specimens; NMHW 2017/0033/0026 (Fig. 4i), NMHW 2017/0033/0027 (Fig. 4j), NMHW 2017/0033/0028a (Fig. 4k), NMHW 2017/0033/0028b (Fig. 4l + seven spec.).

*Measurements.* largest (incomplete) specimen: height: 56 mm, diameter: 47 mm.

*Description.* Large, solid, broad conical shells; apical angle ranging around 35°–40° (note that the specimen illustrated as Fig. 4i is compressed). Protoconch and early teleoconch whorls unknown. Spire whorls low; sculpture consisting of a weak adsutural spiral cord with small, slightly spirally elongated beads at the lower suture adjoined by a more prominent spiral cord of densely spaced, rounded beads. Adapically follow two very prominent, smooth spiral cords with flat backs, separated by a very deep groove, being slightly narrower than spiral cords. Suture deeply canaliculate. Therefore, the lowermost spiral cord of beads is usually hidden in the sunken suture. Last whorl broad, barrel-shaped, somewhat gradate and probably with nodes on upper spiral cord, but the poor preservation obscures most features. Aperture and columellar structure not preserved in the available material.

*Remarks.* This conspicuous species was described by Archiac and Haime (1853) based on a subadult (or incomplete) specimen but was not recorded by Vredenburg (1925, 1928). At Irer, however, the species is not rare in the mudflat deposits. Interestingly, most specimens were settled by oysters.

Eames (1950) recognized that *Cerithium subtrochleare* was preoccupied and proposed *pakistanicum* as new name. The broad conical outline, canaliculate suture and extremely prominent spiral cords suggest a placement in the extinct genus *Campanilopsis* Chavan, 1949. As names ending in -*opsis* are feminine, the correct combination is *Campanilopsis pakistanica*. This genus was described so far only from the Eocene and Oligocene of France and Italy (Chavan 1949; Lozouet 1986; Reid et al. 2008), being represented by the Eocene *C. lemniscata* (Brongniart, 1823) and the Oligocene *C. ceres* (Orbigny, 1852). The type species *Campanilopsis ceres* (=*Telescopium decorticatum* Chavan, 1943) differs from the Indo-West Pacific Miocene species in its beaded spiral cords and less exaggerated suture.

*Stratigraphic and geographic distribution.* Burdigalian of Irer in Iran (Makran), lower Miocene Gaj beds of the Hala Range and Sindh in Pakistan and lower to middle Miocene parts of the Pegu Group in Myanmar (Eames 1950).

Genus ***Vicarya*** Archiac and Haime, 1853

*Type species. Nerinea verneuili* Archiac, 1851; by monotypy. Miocene, Pakistan.

***Vicarya verneuili*** (Archiac, 1851)

Figure 4m

*1851 [*Nerinea*]? *Verneuili* D’Arch. Archiac: p. 286.

1853 *Vicarya verneuili* d’Arch.—Archiac and Haime: p. 298, pl. 28, Figs. 4a–b.

1986 *Vicarya verneuili* (Archiac, 1851)—Kanno: p. 37 (pars), pl. 1, Figs. 1a–b (Pakistani specimen only).

2014 *Vicarya verneuili* (Archiac, 1850)—Jain: p. 129, pl. 28, Figs. 30–31, pl. 38, Figs. 18–19.

*Material.* One fragmentary specimen.

*Measurements*. diameter: 21.2 mm (NMHW 2017/0033/0029).

*Description.* A single spire fragment consisting of two whorls is available; whorls straight-sided, suture weakly incised. Sculpture consisting of a spiral row of very wide spaced nodes placed distinctly below upper suture. Below follow two spiral cords of densely-spaced, slightly axially elongate beads, separated by smooth interspaces of approximately same width. A third spiral cord at the lower suture is largely covered by the next whorl.

*Remarks.* The fragmentary preservation does not allow a clear identification. The general sculpture, however, agrees well with *Vicarya verneuili* from the lower Miocene Gaj beds in Pakistan. *Campanile* species from the lower Miocene Gaj beds in India and Pakistan, described by Archiac and Haime (1853) and Vredenburg (1928) as *Cerithium helli* Archiac and Haime 1853 and *Telescopium charpentieri* (Basterot 1825) differ in their much more prominent sculpture and the adsutural position of the upper spiral cord of nodes. The adsutural row of spines separate also the early and middle Miocene specimens from the Philippines and Indonesia, described by Kanno (1986) and Kase (2008) as *Vicarya verneuili*, from the species from the Pakistani Gaj beds.

*Stratigraphic and geographic distribution.* Burdigalian of Irer in Iran (Makran) and lower Miocene Gaj beds of the Hala Range in Sindh (Pakistan) (Archiac and Haime 1853) and east of Bamnasa in Gujarat (India) (Jain 2014).

Superfamily **Cerithioidea** Fleming, 1822

Family **Cerithiidae** Fleming, 1822

Genus ***Cerithium*** Bruguière, 1789

*Type species. Cerithium adansonii* Bruguière, 1792; subsequent designation by Melville (1978), ICZN Opinion 1109. Recent, Indo-West Pacific.

***Cerithium*** sp.

Figure 4n–o

*Material.* Nine specimens, NMHW 2017/0033/0030 (Fig. 4n), NMHW 2017/0033/0031 (Fig. 4o), NMHW 2017/0033/0032 (seven spec.).

*Measurements.* height: 28.1 mm (incomplete), diameter: 13.1 mm.

*Description:* Medium sized, slender pupoid shells with moderately incised suture; protoconch and early teleoconch not preserved. Teleoconch whorls with slight concavity close below upper suture and moderately convex below. Spire whorls with four spiral rows of prominent nodes; a weak secondary spiral cord with faint beads intercalated between lower two primary cords. Upper spiral cord of beads slightly more prominent and separated from lower cords by deeper groove. Number of beaded primary cords rises to five on penultimate whorl with a nearly smooth, slightly wavy secondary cord between each pair of primary spiral cords. Base rapidly contracting, convex, covered by five wavy spiral cords. Aperture probably wide but largely missing; posterior canal deep, limited by weak parietal denticle. Inner lip moderately broad, reflected, well demarcated from base. Each whorl bears a prominent varix.

*Remarks.* The extant South African *Cerithium crassilabrum* Krauss 1848 is somewhat reminiscent concerning sculpture but differs in its less contracting base and the weaker varices. *Cerithium dolfusi* (Martin, 1916), from the lower Miocene of Indonesia, is also reminiscent of the Iranian species but differs in the incised suture and the beaded spiral cords on the base and the weaker varices [note that Martin (1916) used *dolfusi* in his description and in the figure caption of *Potamides* (*Terebralia*) *Dolfusi*, instead of *dollfusi,* which would be the correct spelling]. This species seems to represent a new species but the available specimens are too fragmentary to serve as types.

*Stratigraphic and geographic distribution.* Only known from the Irer section in Iran, where it was found within the coral reef unit.

Family **Turritellidae** Lovén, 1847

Genus ***Zaria*** Gray, 1847

*Type species*. *Turbo duplicatus* Linnaeus, 1758; by subsequent monotypy. Recent, Indo-West Pacific.

***Zaria angulata*** (Sowerby, 1840)

Figure 4p

*1840 *Turritella angulata* Sowerby: explanation of plates (no page number), pl. 26 Fig. 7.

1853 *Turritella angulata* Sowerby—Archiac and Haime: p. 294, pl. 27 Figs. 6–9.

2009 *Zaria angulata* (Sowerby, 1840)—Harzhauser et al.: p. 343, Fig. 3a–d.

2010 *Zaria angulata* (J. de C. Sowerby, 1840)—Kulkarni et al.: p. 314, Fig. 2g.

2014 *Zaria angulata* (J. de C. Sowerby, 1840)—Jain: p. 134, pl. 27, Figs. 12–19.

*Material.* Two fragmentary specimens (NMHW 2017/0033/0033).

*Measurements.* maximum diameter: 12.6 mm.

*Remarks.* Due to the fragmentary preservation, the identification remains debatable. The wide apical angle and the very prominently keeled angulation, however, agree fully with Indian specimens described by Jain (2014) and support the identification.

*Stratigraphic and geographic distribution. Zaria angulata* (Sowerby, 1840) is documented from the lower Miocene of the Kutch Basin and the Sindh region in Pakistan (Jain 2014; Vredenburg 1928). The westernmost occurrence is known from the Aquitanian of the Qom Basin in Iran (Harzhauser et al. 2002).

Order **Littorinimorpha** Golikov and Starobogatov, 1975

Superfamily **Stromboidea** Rafinesque, 1815

Family **Rostellariidae** Gabb, 1868

Genus ***Tibia*** Röding, 1798

*Type species. Murex fusus* Linnaeus, 1758; subsequent designation by Dall (1906). Recent, Indo-West Pacific.

***Tibia*** cf. ***indica*** Dey, 1961

Figure 5a

cf. *Tibia indica Dey: p. 66, pl. 7, Figs. 1, 7.

*Material.* Two fragmentary specimens (NMHW 2017/0033/0034).

*Measurements.* maximum diameter: 32.4 mm.

*Description.* Medium-sized *Tibia* with weakly convex spire whorls and a moderately convex last whorl with slightly gradate transition into the spire. Inner lip strongly thickened, broad, well demarcated from base. Posterior canal short, narrow, with dorsally twisted tip, terminating distinctly below upper suture. Last whorl with weak shoulder close to the aperture.

*Remarks.* Although the fragmentary preservation does not allow a clear identification, the strongly thickened callus of the inner lip and the short, twisted posterior canal are reminiscent of *Tibia indica*. A difference is the larger size of the Iranian shells and the slightly shorter posterior canal, which reaches up to the suture of the penultimate whorl. The late Miocene *Tibia verbeeki* (Martin 1899), from Indonesia, develops an identical posterior canal and agrees in size but seems to differ in its more convex last whorl (see Leloux and Wesselingh 2009).

*Stratigraphic and geographic distribution. Tibia indica* was originally described from the Burdigalian of Kerala in SW-India (Dey, 1961). Additional occurrences were reported as *Tibia indica* from the lower Miocene of Gujarat in NW-India by Kulkarni et al. (2010) and Jain (2014). The preservation of the illustrated specimens, however, does not allow a clear identification. At the Irer section in Iran, *Tibia* cf. *indica* is not rare in the sandy marls underlying the reef carbonates.

Superfamily **Velutinoidea** Gray, 1840

Family **Triviidae** Troschel, 1863

Genus ***Trivellona*** Iredale, 1931

*Type species. Trivellona excelsa* Iredale, 1931; by monotypy. Recent, West Pacific.

***Trivellona makranica*** Harzhauser sp. nov.

Figure 5c

*Etymology.* Referring to the Makran region.

*Holotype.* One specimen (NMHW 2017/0033/0035), height: 17.5 mm, diameter: 11.3 mm.

*Locality and horizon.* Close to Irer village (N 26°40′55.26′’, E 057°56′1.72′’), Makran, Islamic Republic of Iran; reefal limestone of the Band-e-Chaker Formation, Burdigalian, early Miocene.

*Diagnosis.* Large elongate ovoid *Trivellona* with trapezoid profile and 12 × 2 prominent, rarely bifurcating dorsal ribs. Sharply edged labral shoulder coinciding with axial ridge forming beads at intersections with dorsal ribs. Central, nearly straight aperture with prominent denticles.

*Description.* Shell elongate ovoid with subparallel margins. Dorsal profile trapezoid, moderately convex; dorsal sulcus indistinct, narrowly depressed. Sculpture consisting of 12  × 2 prominent, regular, subparallel and rarely bifurcating dorsal ribs (excluding terminal ribs), separated by broader interspaces; very weak secondary ribs are occasionally intercalated in the central part of the dorsum. Tips of primary ribs along dorsal sulcus only weakly out of phase in central part but slightly more so on terminal tips. Labral shoulder sharply edged coinciding with axial ridge forming beads at intersections with dorsal ribs. A second axial ridge occurs in the slightly angular ventral margin. Aperture in ventral center, very narrow, nearly straight. Labrum moderately convex to slightly flattened, declivous towards aperture throughout with 19 knobby labral denticles; parietal lip angularly edged with 17 columellar teeth projecting into aperture as prominent denticles. Posterior terminal tip blunt, anal canal short, narrow; anterior tip weakly protruding with short, narrow, twisted siphonal canal (terminology follows largely Fehse 2015).

*Remarks.* The Iranian Miocene species is characterized by its large size, cylindrical outline, blunt terminations, knobby denticles and prominent axial ridges at labral shoulder and ventral margin. These features distinguishes the Iranian shell from the three Miocene *Trivellona* species listed by Fehse (2002): *Trivellona coxi* (Schilder, 1936), from the Gaj beds of Pakistan, is highly reminiscent of the Iranian species, but is much smaller and differs in its more spherical outline, the wider aperture and continuous dorsal ribs. *Trivellona ijzermani* (Schilder, 1937), from the early Miocene of Indonesia, is clearly distinguished by its much smaller size and globular shape. *Trivellona shimajiriensis* (MacNeil, 1961), from the Miocene of Japan, differs in its spherical outline and the much narrower labium.

Only few additional triviid species of other genera are described so far from the Miocene of the Indo-West Pacific regions (Fehse 2002). Of these, “*Trivia” subglobosa* Jain, 2014, from the lower Miocene of Gujarat in India, differs in its broad-ovoid outline. *Trivirostra oryza* (Lamarck 1810), from the upper Miocene of Fiji and the Marshall Islands (Ladd 1977), is much smaller, has a deep dorsal sulcus and is ovoid in outline. Similarly, *Dolichupis smithi* (Martin, 1884), from the Miocene of Indonesia, differs obviously from the Iranian shell in its ovate outline and protruding terminal tips (see Leloux and Wesselingh 2009). *Cleotrivia berauensis* (Schilder, 1941), from the Miocene of Borneo, has a spherical outline and a broad dorsal sulcus, *Cleotrivia lodanensis* (Schilder, 1937), from the Miocene of Indonesia, has a spherical outline and *Trivirostra javana* (Schilder, 1937), from the Miocene of Indonesia, has a much broader dorsal sulcus.

*Stratigraphic and geographic distribution.* Only known from the type locality.

Superfamily **Tonnoidea** Suter, 1913

Family **Cassidae** Latreille, 1825

Subfamily **Phaliinae** Beu, 1981

Genus ***Semicassis*** Mörch, 1852

*Type species. Cassis japonica* Reeve, 1848; subsequent designation by Harris (1897). Recent, Japan.

***Semicassis*** cf. ***bisulcata*** (Schubert and Wagner, 1829)

cf. *1829 *Cassis bisulcata* Schubert and Wagner: p. 68, Figs. 3081, 3082.

cf. 2005 *Semicassis bisulcata*—Beu: p. 51, Figs. 106–134 (cum syn.).

*Material.* One partial silicone cast (NMHW 2017/0033/0036).

*Measurements.* height: c. 23 mm, diameter: c. 21 mm.

*Remarks.* Only a silicone cast of the dorsal part of the shell is available showing prominent spiral sculpture and granulose spiral cords on the spire whorls. Despite the fragmentary preservation, the specimen agrees well with strongly sculptured specimens of *Semicassis bisulcata* as reviewed by Beu (2005).

*Stratigraphic and geographic distribution. Semicassis bisulcata* is a widespread extant Indo-West Pacific species, which appears already during the early and middle Miocene, when it is recorded from Tanzania (Harzhauser 2009), Kerala in SW-India (Dey 1961) and Indonesia (Beu 2005). Therefore, the occurrence of *Semicassis* cf. *bisulcata* in the Burdigalian of Makran would fit in the Miocene distribution pattern of *Semicassis bisulcata*.

Order **Neogastropoda** Wenz, 1938

Superfamily **Buccinoidea** Rafinesque, 1815

Family **Melongenidae** Gill, 1871 (1853)

Genus ***Melongena*** Schumacher, 1817

*Type species. Melongena fasciata* Schumacher, 1817 (= *Murex melongena* Linnaeus, 1758); by monotypy. Recent, Caribbean.

***Melongena*** cf. ***lainei*** (Basterot 1825)

Figure 5b

cf. *1825 *Pyrula Lainei* Basterot: p. 67, pl. 7, Fig. 8.

cf. 2001 *Melongena lainei* (Basterot 1825)—Lozouet et al.: p. 62, pl. 28, Figs. 1–3 (cum syn.).

cf. 2007 *Melongena lainei* (Basterot 1825)—Harzhauser: p. 106, pl. 6, Fig. 3 (cum syn.).

*Material.* One specimen (NMHW 2017/0033/0037).

*Measurements.* height: 78 mm, diameter: 58 mm.

*Remarks.* A single poorly preserved specimen is available with remnants of sculpture on the spire. Outline and the characteristic very coarse and prominent spiral cords on the low angled sutural ramp agree fully with the Western Tethyan/Proto-Mediterranean *Melongena lainei.*

*Stratigraphic and geographic distribution. Melongena lainei* is widespread during the Chattian to Burdigalian in the Western Tethys/Proto-Mediterranean and the Paratethys Sea (Lozouet et al. 2001). *Melongena* cf. *lainei* was recorded in the Proto-Indian Ocean from the Chattian of the Sultanate of Oman (Harzhauser 2007) and the lower Miocene of the Iranian Makran (this paper), Sindh in Pakistan and Kutch in NW-India (Vredenburg 1924, 1925; Kulkarni et al. 2010).

Superfamily **Muricoidea** Rafinesque, 1815

Family **Volutidae** Rafinesque, 1815

Subfamily **Athletinae** Pilsbry and Olsson, 1954

Genus ***Athleta*** Conrad, 1853

Subgenus ***Volutospina*** Newton, 1906

*Type species. Conus spinosus* Linnaeus, 1758; by original designation. Middle Eocene, England.

***Athleta*** (***Volutospina***) ***sykesi*** (Archiac and Haime 1853)

Figure 5d

*1853 *Voluta sykesi* Archiac and Haime: p. 324, pl. 32, Fig. 3–3a.

1925 *Athleta* (*Volutospina*) *dentata* var. *sykesi* Archiac and Haime—Vredenburg: p. 133.

2014 *Athleta* (*Volutospina*) *sykesi* (Archiac and Haime 1853)—Jain: p. 184, pl. 35, Figs. 17–21.

*Material.* One specimen (NMHW 2017/0033/0038).

*Measurements.* height: 29.2 mm, diameter: 16.1 mm.

*Description.* Small, moderately slender volutid; spire comprising five whorls; early spire conical, forming an angle of c. 80°, later coeloconoid. Sculpture consisting of two rows of small spiny nodes along the shoulder adjoined by two weak spiral threads on sutural ramp. Penultimate and last whorls slightly widening with concave sutural ramp. Upper spiral row of nodes fades out on last whorl, which bears two narrow, smooth spiral cords on concave part of ramp and 24 spiny nodes along shoulder. Below shoulder appear broad axial swellings, crossed by broad spiral cords separated by slightly narrower interspaces; base weakly constricted. Aperture covered by sediment.

*Remarks.* Harzhauser et al. (2009) listed this species as synonym of *Athleta* (*Volutospina*) *dentata* (Sowerby, 1840). As pointed out by Jain (2014); however, the smaller size, slender outline and higher spire allow a separation of *Athleta* (*Volutospina*) *sykesi* from *Athleta* (*Volutospina*) *dentata.* In addition, the higher number of spiny nodes distinguishes *A.* (*V.*) *sykesi* from *A.* (*V.*) *dentata* (see also description in Vredenburg 1925).

*Stratigraphic and geographic distribution.* Burdigalian of the Iranian Makran (this paper) and lower Miocene Gaj beds of Sindh in Pakistan and Gujarat in NW-India (Jain, 2014).

Subclass **Heterobranchia** Burmeister, 1837

Order **Cephalaspidea** Fischer, 1883

Superfamily **Philinoidea** Gray, 1850 (1815)

Family **Scaphandridae** Sars, 1878

Genus ***Scaphander*** Montfort 1810

*Type species. Bulla lignaria* Linnaeus, 1758, by original designation. Recent, Mediterranean Sea and North Eastern Atlantic.

***Scaphander javanus*** (Martin, 1879)

Figure 5e

1840 *Bulla lignaria* Linn.—Sowerby: explanation of plates (no page number), pl. 26 Fig. 1 [non *Scaphander lignarius* (Linnaeus, 1758)].

*1879 *Bulla* (*Scaphander*) *javana* Martin: p. 85, pl. 13, Fig. 21.

1925 *Scaphander javanus* Martin—Vredenburg: p. 11.

2009 *Scaphander javanus* (Martin, 1879)—Leloux and Wesselingh: p. 41, pl. 81, Fig. 15.

2010 *Scaphander javanus* Martin–Kulkarni et al.: p. 340.

2014 *Scaphander javanus* Martin, 1879—Jain: p. 206, pl. 38, Figs. 16–17.

*Material.* One specimen (NMHW 2017/0033/0039).

*Measurements.* height: 31 mm, diameter: >14 mm.

*Remarks.* A large *Scaphander* species is abundant in fine-sandstone beds underlying the Irer reef carbonates. The preservation is always poor due to dissolution and only a single cast of a ventral part could be partly extracted from the sandstone. The sculpture of the specimen consists of > 70 densely spaced spiral cords, which tend to bifurcate in the anterior and posterior parts of the shell, separated by narrow, punctate interspaces. This species is frequently reported from the early Miocene *Western Indian Province* from Gujarat and Kerala in India as *Scaphander javanus* (Martin, 1879) (e.g., Dey 1961; Jain 2014). The Neogene Indonesian species agrees in general shape, size and sculpture but is based on a moderately preserved specimen. Therefore, the identification of the Indian, Iranian and Pakistani specimens remains debatable.

*Stratigraphic and geographic distribution.* Neogene of Indonesia (Leloux and Wesselingh 2009); early Miocene of Kutch and Kathiawar (Sowerby 1840; Jain 2014), middle Miocene of Kathiawar (Jain 2014), early Miocene of Kerala (Dey 1961).

## Discussion and conclusions

### Paleoecology

Molluscs are generally rather rare in the Irer section, and the described specimens were collected during several days of intense field work. No mass occurrences or gastropod coquinas were detected. The specimens are usually preserved as calcitic pseudomorphs and as internal casts; the taphonomic bias is considerable. Nevertheless, the species form three distinct assemblages, which derive from three distinct litho-facies without overlapping occurrences:

1. The first group comprises *Neritopsis gedrosiana* sp. nov., *Angaria* cf. *delphinus* (Linnaeus, 1758), *Calliostoma irerense* sp. nov., *Calliostoma mohtatae* sp. nov., *Turbo* cf. *petholatus* Linnaeus, 1758, *Cerithium* sp. and *Trivellona makranica* sp. nov. These taxa were collected partly in situ within the reef carbonates and partly in the associated scree. Especially in the reef carbonates, a strong taphonomic bias can be assumed. Vetigastropods and neritimorph with nacreous shell layers (Mutvei 1978; Hedegaard 1997) are clearly over-represented compared to other groups with less resistant shells, such as various cypraeids, muricids and fasciolariids, which are documented by internal casts, but lack diagnostic features. The reef-associated assemblage is strongly dominated by *Calliostoma mohtatae*, which is by far the most abundant mollusc in the section. All other taxa are recorded by few specimens only (e.g., *Cerithium* sp., *Neritopsis gedrosiana* and *Calliostoma irerense*) or were found as singletons (*Angaria* cf. *delphinus, Trivellona makranica*).
  Extant *Neritopsis* species are exclusively found in reefal environments and submarine caves (Kano and Kase 2008) but empty shells may also appear on the reef slope (Zuschin et al. 2009). *Turbo petholatus* is common in coral reefs, found mainly between 10 and 40 m water depth (Kreipl and Alf 2008). Similarly, *Angaria delphinus* prefers rocks and rocky parts of coral reefs (Tan and Low 2013; Sanpanich and Duangdee 2015). Extant *Trivellona* species prefer deeper water from 20 m down to bathyal settings (Fehse 2002). They settle fine to coarse sand, coral rubble and even rocky substrates (Fehse 2002). These data suggest a setting in roughly 20 m water depth for the reef, with ample crevices. This interpretation is in line with the interpretation of the coral assemblage of the Irer reef by Ghaedi et al. (2016). According to these authors, the reef was dominated by platy and foliaceous coral taxa, which are typical for mesophotic to euphotic conditions with low to moderately high water energy in several tens of meters water depth.
2. The second group consists of *Cernina carlei* (Finlay, 1927), *Zaria angulata* (Sowerby, 1840), *Tibia* cf. *indica* Dey, 1961, *Semicassis* cf. *bisulcata* (Schubert and Wagner, 1829), *Athleta* (*Volutospina*) *sykesi* (Archiac and Haime 1853) and *Scaphander* cf. *javanus* (Martin, 1879). These specimens were collected from calcareous marls and argillaceous limestones underlying the Irer reef and from sandstones, exposed below the measured section. The preservation is poor and the shells are frequently dissolved. The herbivorous extant *Cernina fluctuata* occurs in shallow marine habitats but was also dredged from 100–150 m water depth (Hollman 2008); its Miocene pendant *Cernina carlei* was documented from seagrass-associated assemblages from Kerala in SW-India (Harzhauser 2014). The extant *Semicassis bisulcata* is a mainly subtidal species on sand or mud (Robba et al. 2007). Similarly, *Zaria angulata* is found in shallow subtidal assemblages in Kutch (Harzhauser et al. 2009). *Scaphander* is a predominantly deep-sea genus, but several species occur at shallower depths below c. 20 m (Eilertsen and Malaquias 2015). All these gastropods, along with several venerid bivalves and rare clypeasteroid echinoderms, lived at least partly buried in the sediment. Hence, despite the poor preservation, the assemblage points to 20 m to 150 m deep subtidal clayey/sandy soft-bottom environments, settled by infaunal benthos.
3. The third group comprises mainly potamidid gastropods, such as *Terebralia gajensis* Vredenburg, 1928, *T. miosulcata* Vredenburg, 1928 and *T. sublignitarum* Vredenburg, 1928, along with *Campanilopsis pakistanica* (Eames, 1950) and *Vicarya verneuili* (Archiac, 1851), which belong to extinct genera. The buccinoid *Melongena* cf. *lainei* (Basterot 1825) is the only non-potamidid in the assemblage. The specimens were collected from dark brown clayey marls. Wave ripples and root traces are typical in these deposits, suggesting a littoral mudflat depositional environment. This interpretation is fully supported by the diverse potamidid assemblage. *Terebralia* species are highly correlated with mudflats and mangrove forests (Houbrick 1991; Pape et al. 2008). Some (if not all) extant *Terebralia* species seem to feed almost exclusively on mangrove leaves and propagules (Hogarth 2015). Similarly, *Telescopium*, which is probably closely related with the extinct *Campanilopsis,* is an obligately mangrove-associated gastropod (Houbrick 1991; Reid et al. 2008). The extinct *Vicarya* is interpreted by Kanno (1986) as mangrove-associated and *Melongena* and allied genera are also frequently found in mangrove systems and various shallow marine habitats with low water energy (Vermeij and Raven 2009). Hence, the species of assemblage three point to the presence of mangrove forests. Potamidids, however, tend to form mass occurrences within their habitat; this contrasts with the rather scattered occurrence of shells in the Burdigalian mud flat deposits. Moreover, many *Terebralia* shells are partly abraded and all *Campanilopsis* shells were found with attached oysters. Therefore, the shells might have been inhabited by hermit crabs. Shells of *Telescopium* and *Terebralia* are both well known to be intensively pagurized (Wells 2003; Epa and de Silva 2011; Willan 2013; Kihia et al. 2015). This mechanism would have promoted transport of potamidid shells far beyond their preferred mangrove environment across the vast tidal mud flat of the northern Indian Ocean.

### Paleobiogeography

The documented assemblages show a two fold biogeographic pattern. The mudflat-associated assemblage and the subtidal soft-bottom assemblage are typical elements of the Gaj-type fauna as described by Vredenburg (1925, 1928). Most species are reported from the lower Miocene Gaj beds of Pakistan; e.g., *Terebralia gajensis* Vredenburg, 1928, *Zaria angulata* (Sowerby, 1840), *Athleta* (*Volutospina*) *sykesi* (Archiac and Haime 1853). *Terebralia miosulcata* Vredenburg, 1928 and *T. sublignitarum* Vredenburg, 1928 were known so far only from the middle to upper Miocene Talar beds. This group of species can thus be considered as typical representatives of the *Western Indian Province* (WIP) sensu Harzhauser (2007). The faunistic relation to the *Mediterranean*-*Iranian Province* (MIP) in the Proto-Mediterranean Sea (Harzhauser et al. 2002) is very low. A single species of this group, *Melongena* cf. *lainei* (Basterot 1825), is also (or mainly) known from coeval deposits of the Proto-Mediterranean and eastern Atlantic (Lozouet et al. 2001). Although the preservation of the available specimen is poor, occurrences mentioned by Vredenburg (1925) from Pakistan, seem to prove its presence in the WIP.

Some taxa represent morphological sibling WIP/MIP species pairs: *Terebralia sublignitarum* Vredenburg, 1928 (WIP) versus *T. duboisi* (Hörnes, 1855) (MIP) and *Terebralia gajensis* Vredenburg, 1928 (WIP) versus *T. lignitarum* (Eichwald, 1830) (MIP). Another example might be represented by *Melongena lainei* (Basterot 1825) in Europe versus *M*. cf. *lainei* in the Proto-Indian Ocean. These examples may represent cases of vicariance, documenting the already established biogeographic and genetic separation between closely related taxa in the MIP and WIP.

The group of well-known WIP elements from the mudflats and subtidal soft-bottom environments of the northern Indian Ocean is contrasted by a species group, which is unknown from other localities. This species group comprises exclusively species, which were found within the reef carbonates. Such Miocene carbonates, however, are rare along the coasts of eastern Iran, Pakistan and India in the western part of the Indian Ocean (McCall et al. 1994; Clift et al. 2008). Reef growth was effectively suppressed by an enhanced supply of siliciclastic sediment to the northern Indian Ocean due to the rise of the Himalayas and the Tibetan Plateau (Chatterjee et al. 2013) and the transformation of the Asian climate from a zonal to a monsoon-dominated pattern (Guo et al. 2008; Reuter et al. 2013). Therefore, it is little surprising that the reef-associated species from the Irer section are largely new records. In addition, *Angaria* cf. *delphinus* (Linnaeus, 1758) and *Turbo* cf. *petholatus* Linnaeus, 1758 are probably conspecific with extant Indo-West Pacific species, although the identifications are arguable due to the poor preservation. A typical element of the modern Indo-West Pacific Region is also the triviid genus *Trivellona* (Fehse 2002, 2015). Therefore, the coral-associated assemblage can also be interpreted as a typical WIP element, lacking any relation to the *Mediterranean*-*Iranian Province.* The faunistic relation with the coeval Proto-Indo-Polynesian Province is also surprisingly low; only *Scaphander javanus* seems to occur in both provinces, but the status of this species is unclear (see above).

Thus, the early Miocene gastropod assemblages from the northern Indian Ocean are clearly part of the Western Indian Province of the later Indo-West Pacific Region. Despite its close position to the junction to the Proto-Mediterranean Sea or the *Gomphotherium*-landbridge, respectively, the faunistic influence from the west is negligible. These results coincide with those on corals and foraminifers by McCall et al. (1994).
